# Research on construction method and validity mechanism of robust analysis model in snow peach quality detection based on visible-near infrared spectroscopy

**DOI:** 10.3389/fnut.2022.1042868

**Published:** 2022-10-18

**Authors:** Yong Hao, Xiyan Li, Chengxiang Zhang, Zuxiang Lei

**Affiliations:** ^1^School of Mechatronics and Vehicle Engineering, East China Jiaotong University, Nanchang, China; ^2^Key Laboratory of Conveyance Equipment of the Ministry of Education, East China Jiaotong University, Nanchang, China; ^3^School of Civil Engineering and Architecture, East China Jiaotong University, Nanchang, China

**Keywords:** robust model, Sparse Partial Robust M Regression, visible-near infrared reflectance spectroscopy, soluble solids content, Lijiang snow peach

## Abstract

Visible-near infrared (Vis-NIR) spectra analysis method is widely used in the quality grading of bulk fruits with its rapid, non-destructive, diverse detection modes and flexible modular integration scheme. However, during the online grading of fruits, the random mechanized way of dropping fruit onto the conveyor belt method and the open detection environment led to more spectral abnormal samples, which affect the accuracy of the detection. In this paper, the soluble solids content (SSC) of snow peach is quantitatively analyzed by static and online detection methods. Several spectral preprocessing methods including Norris-Williams Smoothing (NWS), Savitzky-Golay Smoothing (SGS), Continuous Wavelet Derivative (CWD), Multivariate Scattering Correction (MSC), and Variable Sorting for Normalization (VSN) are adopted to eliminate spectral rotation and translation errors and improve the signal-to-noise ratio. Monte Carlo Uninformative Variable Elimination (MCUVE) method is used for the selection of optimal characteristic modeling variables. Partial Least Squares Regression (PLSR) is used to model and analyze the preprocessed spectra and the spectral variables optimized by MCUVE, and the effectiveness of the method is evaluated. Sparse Partial Least Squares Regression (SPLSR) and Sparse Partial Robust M Regression (SPRMR) are used for the construction of robust models. The results showed that the SGS preprocessing method can effectively improve the analysis accuracy of static and online models. The MCUVE method can realize the extraction of stable characteristic variables. The SPRMR model based on SGS preprocessing method and the effective variables has the optimal analysis results. The analysis accuracy of snow peach static model is slightly better than that of online analytical model. Through the test results of the PLSR, SPLSR and SPRMR models by the artificially adding noise test method, it can be seen that the SPRMR method eliminates the influence of abnormal samples on the model during the modeling process, which can effectively improve the anti-noise ability and detection reliability.

## Introduction

Lijiang snow peach is an excellent variety of winter peaches cultivated by Lijiang's unique natural environment, with an average single fruit of about 500 g. With a regular shape, large fruit and bright color, each peach is rich in nutrients (1). Proper consumption of snow peach can play a role in tonifying qi and moistening lungs, and can prevent cardiovascular diseases as well. As a relatively high-end new fruit in China, snow peach is deeply loved by consumers. In recent years, with the gradual enhancement of health awareness, consumers have higher requirements for the taste and internal quality of snow peach. As a result, fruit producers and retailers must strictly control the quality of fruit. The internal SSC of snow peach characterizes its sweetness. The higher the sweetness, the better the taste. However, there is no correlation between sweetness and appearance quality. Although the fruit industry is the third largest planting industry in China, its share of international trade has been low. The main reason is that the post-harvest processing ability of the fruit is low. Therefore, it is important to explore a non-destructive, green and rapid detection method. The agricultural standard NYT 2026–2011 (Evaluation Standard for Excellent Crop Germplasm Resources Peach) stipulates that Soluble Solids Content (SSC) is one of the important indicators to identify whether the peach is an excellent germplasm resource (2). The commercial processing of peach after picking mainly includes the external quality detection of fruit shape index and surface defects based on machine vision and the internal quality grading based on SSC. Due to the regular shapes of snow peach, the detection of SSC has become an important basis for fruit suppliers to judge their quality grades. However, the conventional measurement method of fruit SSC requires the process of sampling, crushing, juicing and filtering, and then the filtrate is detected by digital refractometer. This destructive sampling detection method is not suitable for the commercialization of large-scale fruits.

Visible-near infrared (Vis-NIR) spectroscopy is widely used in the detection of fruit components and defects with its rapid, nondestructive, and flexible integrated detection device. Jiang et al. (3) studied the online detection of SSC in navel orange by using Vis-NIR technology. The results showed that the variable selection method combined with Partial Least Squares Regression (PLSR) can obtain a better analytical model, with Correlation Coefficient of Prediction (*R*_*P*_) and Root Mean Squared Error of Prediction (RMSEP) of 0.824 and 0.670, respectively. Liu et al. (4) used dynamic online detection equipment to collect spectral information to compare the changes of peach SSC under two storage conditions, and obtained *R*_*P*_ of 0.819 and 0.828 for the peach SSC models under room temperature and refrigerated conditions, respectively. Yang et al. (5) combined the Vis-NIR spectroscopy to establish an analytical model for predicting tomato SSC. The results showed that the PLSR model with 22 key wavelengths selected by Competitive Adaptive Weighted Sampling (CARS) had better model performance than the full-wavelength model. Kim et al. (6) discussed the prediction of melon SSC based on different analytical models. The results showed that the prediction accuracy of different modeling methods was quite different, and the best method was PLSR combined with Artificial Neural Networks (ANN). Hao et al. (7) explored the influence of three kinds of navel orange placement postures on the modeling results. The results showed that the spectral information of measured samples under different postures was different, and the placement position affected the prediction accuracy of the model. Because the randomness of sample placement affects the repeatability of spectrum, more spectral abnormal samples are generated, resulting in large prediction error of online model.

In recent years, the construction of the robust regression model has been widely used in many fields. Lu et al. (8) applied Robust Partial Least Squares Regression (RPLSR) and Partial Robust M Regression (PRMR) to determine the content of each component in rapeseed. The results showed that compared with traditional quantitative methods, robust regression could reduce the influence of outliers on the model accuracy and obtain reliable prediction results. Yao et al. (9) utilized Vis-NIR combined with Sparse Partial Least Squares Regression (SPLSR) to construct an industrial analytical model of sawdust biomass. The results showed that SPLSR could ignore the interference of singular values and improve the interpretability of the model. Robust Regression (Rob-Reg) method proposed by Hao et al. (10) could effectively overcome the over-fitting problem, establish a reliable and stable analytical model, and obtain stable prediction results. The above research results show that the robust regression method has the advantages of preventing over-fitting and reducing the influence of outliers or strong influence values on the stability of the model, which can improve the prediction accuracy of the model to a certain extent.

When static and online detection of Lijiang snow peach SSC is carried out by Vis-NIR, the static collection mode of manually placed samples can ensure the repeatability of spectral collection areas and spectra; While during the online detection, the random loading method with a mechanized conveyor belt leads to inconsistent spectral acquisition area and more abnormal spectral samples, thus affecting the accuracy of detection. In this paper, different spectral preprocessing and MCUVE methods are used to enhance and optimize the characteristic variable information of fruit SSC. The robust grading model of snow peach quality is constructed by combining SPLSR and Sparse Partial Robust M Regression (SPRMR), so as to improve the anti-interference and analysis accuracy of the model.

## Materials and methods

### Sample preparation

The samples in this experiment were called Yunnan Lijiang snow peach, which was collected from an orchard and transported to the laboratory under refrigeration. The samples were stored at a constant temperature of 20°C, and the experiments were carried out after standing for 24 h. In order to prevent the stains attached to the surface of the snow peach after harvesting from affecting the accuracy of the spectrum acquisition, it is necessary to clean the snow peach before the experiment. In the experiment, the reflection spectra of 400 samples were collected for static and online detection modes respectively. The Atago digital refractometer (SSC detection, Saitama, Japan) was used to conduct five SSC tests on the filtrate after filtering the whole fruit juice after denucleating, and the average value was used as the final SSC of the samples. The sample set was divided by means of the average distribution of SSC contents combined with the spectral-spatial distribution. Select representative samples for modeling from the aspects of SSC content and spectral distribution, so as to ensure that the modeling samples cover the test samples. The calibration set and the prediction set are divided according to the ratio of 2:1. Finally, 267 calibration samples were used for modeling, and 133 prediction samples for model evaluation. The statistical information of the experimental samples is shown in Table 1.

**Table 1:** Statistical information of the experimental samples.

**Detection mode**	**Sample set**	***N***	***R_SSC_*** **(°Brix)**	**Mean**	**Standard deviation**
Static	Calibration set	267	10.1–15.1	12.859	0.966
	Prediction set	133	10.1–15.0	12.844	0.952
Online	Calibration set	267	10.1–15.1	12.872	0.943
	Prediction set	133	10.1–15.1	12.863	0.935

*N* represents the number of samples*; R*_*SSC*_ indicates the range of SSC.

### Acquisition of Vis-NIR spectra

**Figure 1:**
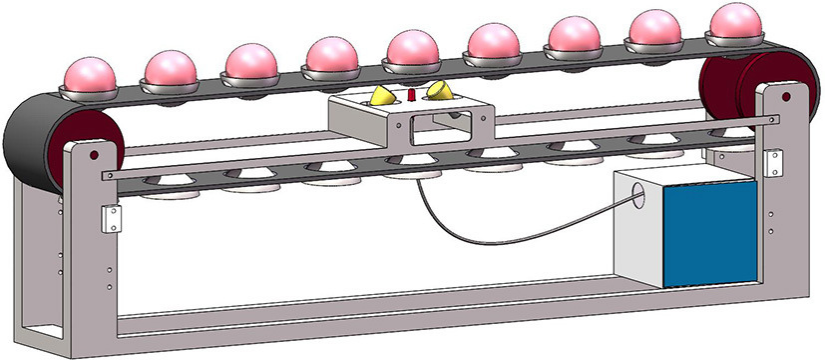
Schematic diagram of spectral acquisition device.

Vis-NIR spectra of snow peach were acquired by Ocean Optic Marine Optical QE65000 spectrometer (Optical inspection, Florida, USA), with a wavelength range of 346–1,130 nm and a resolution of ca. 1 nm. Therefore, each spectrum contains 1,044 wavelength variables. The light source adopted two 100-watt halogen tungsten lamps (OSRAM), which were arranged at an angle of 45 degrees along the transmission direction of the conveyor belt. The schematic diagram of spectral acquisition device is shown in Figure 1, which consists of a spectrometer, a light source, a conveyor belt, an optical fiber and a computer. Both static and online detection experiments were completed on this device, and the conveyor belt stopped during the static collection, and the fruit cup was located directly above the center of two light sources. During the dynamic acquisition, the speed of the conveyor belt was 6 peaches per second. The difference between static detection and online detection is that the static method can control the position of the fruit to obtain more stable spectral information by manually placing the fruit. While the online detection adopts the method of mechanized random fruit loading, and the position of the fruit reaching the detection point is random, thus the obtained spectral information contains more interference.

### Preprocessing of Vis-NIR spectra

In order to eliminate the background and noise existing in the original spectrum and the interference caused by the instruments and placement methods in the acquisition process, it is necessary to preprocess the original spectrum. Data smoothing, derivative and scattering correction are used to preprocess the static and dynamic online spectra (11). The purpose of the smoothing algorithm is to improve the signal-to-noise ratio of the research samples and eliminate spectral noise. The derivative algorithm is mainly to eliminate the influence of the spectral instrument on the signal. The scattering correction is used to eliminate scattering effects caused by particles of different sizes. Among them, Norris-Williams Smoothing (NWS) (7) and Savitzky-Golay Smoothing (SGS) (12) are used for smoothing; the derivative adopts Continuous Wavelet Derivative (CWD) (13) method; Multivariate Scatter Correction (MSC) (14) and Variable Sorting for Normalization (VSN) (15) methods are used for scattering correction.

### MCUVE variable optimization method

The purpose of variable selection is to optimize the model, save calculation time and make the model more explanatory (16). Each Vis-NIR spectrum of peach samples contains 1,044 wavelength points, with many uninformed variables and high-dimensional overlap. In order to eliminate redundant and uninformed variables, this paper adopts MCUVE variable selection method to select effective wavelength.

The idea of MCUVE method is basically consistent with that of Uninformative Variable Elimination (UVE). The difference is that MCUVE uses Monte Carlo (MC) sampling instead of adding random noises to UVE, which combines MC process with UVE, makes full use of the intrinsic correlation between samples, evaluates the contribution of wavelength variables in high-dimensional spectral data, sorts the stability of each wavelength according to the contribution value of each wavelength, and finally establishes a series of the PLSR models. At last, the upper and lower thresholds are determined by the Root Mean Squared Error of Cross Validation (RMSECV) of the optimal PLS model. The variables outside the threshold are retained and those within the threshold are discarded.

### Calibration model

#### Partial least squares regression

Partial Least Squares Regression (PLSR) (17) is a widely used regression technique for Vis-NIR spectral data analysis. This method combines the advantages of principal component regression, multiple linear regression and canonical correlation analysis, and can be applied to an effective regression method for processing high-dimensional data. Its core idea is to project high-dimensional data into the hidden space, obtain the RMSECV value by the leave-one-out method or the MC cross-validation method, and obtain the best factor number combined with *F*-test and establish the model.

#### Robust regression method

The SPLSR method is realized by NIPALS algorithm, which is a linear combination of original variables through a set of weighted vectors, belonging to the sparse version of PLS (18). The algorithm generates sparse solutions by keeping the subsequence structure of the direction vector in the restricted *X* space of the selected variables (19). The SPLSR algorithm is a method that integrates dimensionality reduction and variable selection (18). In general, methods with inherent variable selection properties have smaller prediction errors than methods lacking the inherent variable selection properties. Compared with the classical PLSR algorithm, the SPLSR algorithm can reduce the interference of irrelevant information, improve the accuracy of the quantitative analysis of the model, and enhance the explanatory ability of the model (9).

Based on PRMR algorithm, SPRMR algorithm introduces sparse factors. SPRMR estimator can be regarded as a sparse version of PRMR estimator, and it can also be used as a method of SPLSR estimator. SPRMR is the first method to combine dimension reduction with regression, which can generate partial least squares estimation, sparse and robust to feature vectors and responses. The advantage of SPRMR lies in that the algorithm can not only automatically identify vertical outliers and leverage points (vertical outliers are outliers in the response; lever points are outliers in the predictor), but also automatically reduce the dimension of high-dimensional data. The specific algorithm of PRMR is shown in Reference (19), and SPRMR is shown in Reference (20).

### Model evaluation

Correlation Coefficient of Cross Validation (*R*_*CV*_) and RMSECV are used as evaluation indexes of preprocessing methods and variable selection, and the *R*_*CV*_, *R*_*P*_, RMSECV, RMSEP, Residual Predictive Deviation (RPD) and The Ratio of Error Range (RER) are used as evaluation indexes of regression models. The closer *R*_*CV*_ and *R*_*P*_ are to 1 and RMSEP and RMSECV are to 0, indicating that the better the model is. Among them, the smaller the error between *R*_*CV*_ and *R*_*P*_, RMSECV and RMSEP is, the better the model is, and the smaller the difference is, the more stable the model is. The values of RPD and RER indicate the model quality. The larger the value is, the better the model is. Nicolaï et al. (21) defined that RPD value of 1.5–2 means the model can make rough quantitative prediction; 2–2.5 means the model has reliable prediction performance; >3 means the model has excellent prediction performance; Badaró et al. (22) defined that RER value >10 means the model can achieve excellent prediction accuracy.

The formulas of *R*_*CV*_, *R*_*P*_, RMSEC, RMSEP, RPD and RER are as follows:


(1) $$\begin{array}{l} R_{CV},R_{P}=\sqrt{1-\left[\overset{n}{\underset{i=1}{\sum }}{\left(y_{i}-y_{pi}\right)}^{2}\right]/\left[\overset{n}{\underset{i=1}{\sum }}{\left(y_{i}-y_{m}\right)}^{2}\right]} \end{array}$$

(2) $$\begin{array}{l} RMSECV,RMSEP=\sqrt{\frac{1}{n}\overset{n}{\underset{i=1}{\sum }}{\left(y_{i}-y_{pi}\right)}^{2}} \end{array}$$

(3) $$\begin{array}{l} \begin{array}{l} RPD=\frac{SD}{RMSEP} \end{array}\\ \begin{array}{l} =\sqrt{\frac{1}{n-1}\overset{n}{\underset{i=1}{\sum }}{\left(y_{i}-y_{m}\right)}^{2}}/\sqrt{\frac{1}{n}\overset{n}{\underset{i=1}{\sum }}{\left(y_{i}-y_{pi}\right)}^{2}} \end{array} \end{array}$$

(4) $$\begin{array}{l} RER=P_{r}/RMSEP \end{array}$$

wherein, *y*_*i*_ and *y*_*pi*_ represent SSC values of the calibration set and the prediction set respectively; *y*_*m*_ represents the average value of actual measured SSC in the calibration set and prediction set; *n* represents the number of samples in the calibration set or prediction set; *SD* represents the standard deviation of prediction set; *P*_*r*_ represents the SSC range of the prediction set.

## Results and discussion

### Vis-NIR analysis of snow peach samples with static and online detection

**Figure 2:**
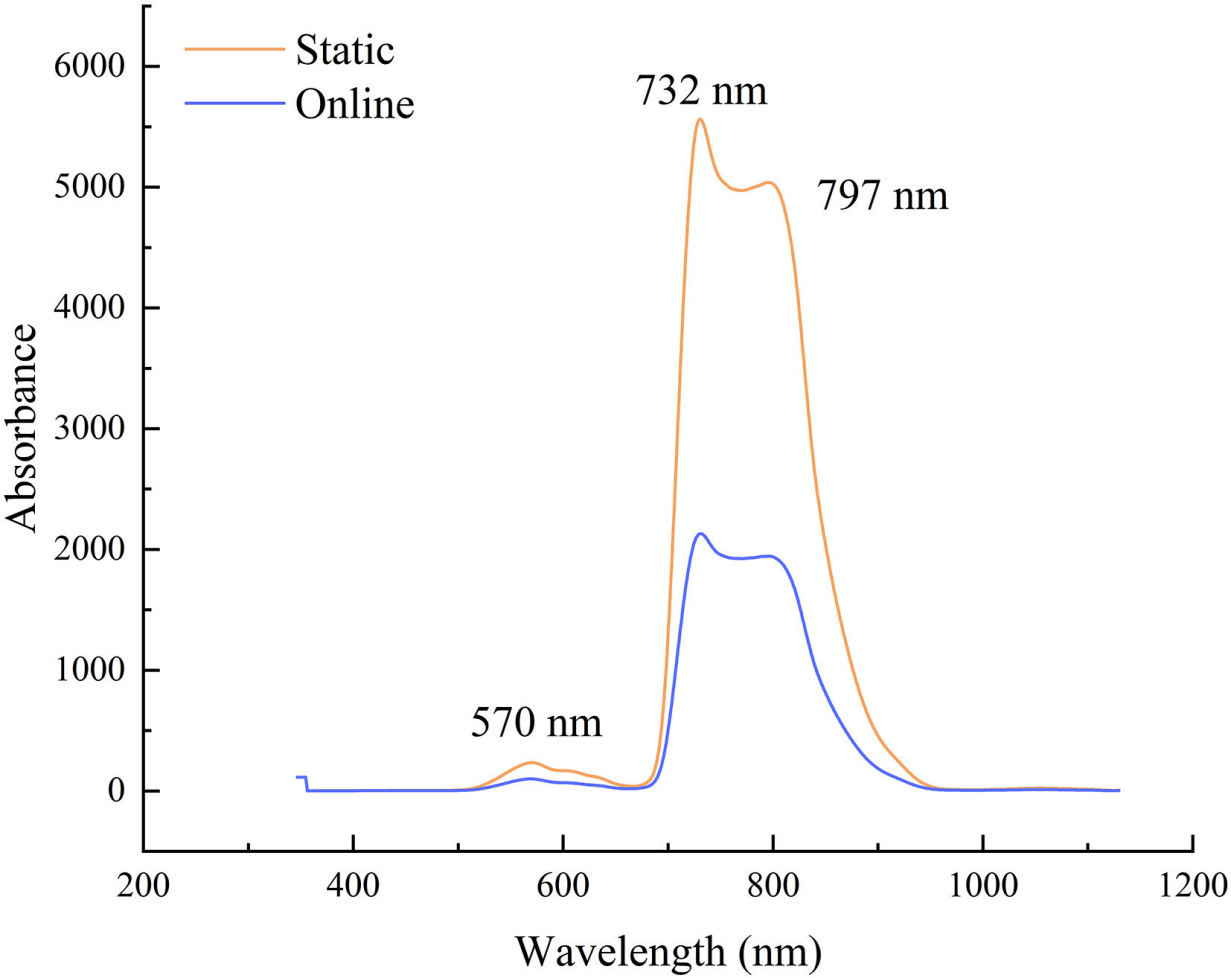
Average Vis-NIR spectra of snow peach samples with static and online detection.

In order to explore the overall difference between static and online detection spectra, the Vis-NIR average reflectance spectra of 400 snow peacher samples were used for illustration (Figure 2). It can be seen from the figure that the spectral curves obtained under different detection modes are basically similar. The difference is that the static spectra have higher response intensity than the online spectra, and spectra have obvious absorption peaks around 570, 732, and 797 nm. The reason for the existence of peaks is related to the stretching and contraction of hydrogen-containing groups (O–H, C–H or N–H) in the internal components of snow peach (23). The two absorption peaks observed around 732 and 797 nm may be related to the stretching of the third overtone of C–H functional group and the second and the third overtones of O–H functional group respectively (24). The absorption peak at 570 nm is mainly related to colors and shapes of the peach (25).

### Spectral preprocessing and characteristic analysis

#### Optimization of spectral preprocessing methods

In order to further improve the quality of the modeling spectra, five different preprocessing methods are used to eliminate the drift, scattering and noises of original spectra. PLSR is used to model and analyze the preprocessed spectra, and the effectiveness of the method is evaluated. The evaluation indicators are LV_S_, *R*_*CV*_ and RMSECV. After sample spectra are preprocessed by different preprocessing methods, the modeling results of snow peach SSC models are shown in Table 2.

**Table 2:** The modeling results of snow peach SSC models with different spectral preprocessing methods.

**Preprocessing methods**	**Static**			**Online**		
	**LVs**	***R_*CV*_***	**RMSECV**	**LVs**	***R_*CV*_***	**RMSECV**
Raw	18	0.831	0.540	19	0.820	0.543
NWS	18	0.824	0.550	19	0.810	0.556
SGS	19	0.991	0.127	20	0.991	0.125
CWD	18	0.820	0.555	19	0.811	0.554
MSC	18	0.827	0.545	18	0.811	0.556
VSN	15	0.822	0.554	18	0.806	0.566

NWS selects 15-point smoothing; in static mode, the polynomial order of SGS is set to 3, and the number of smoothing points is set to 17; in online mode, the polynomial order of SGS is set to 3, and the number of smoothing points is set to 15; the decomposition scale of CWD is set to 60.

It can be seen from Table 2 that the LVS, *R*_*CV*_ and RMSECV of the original static spectral modeling are 18, 0.831 and 0.540, respectively; the LVS, *R*_*CV*_ and RMSECV of the original dynamic spectral modeling are 19, 0.820 and 0.543, respectively. By comparison, it can be seen that, except for the improvement of *R*_*CV*_ and the decrease of RMSECV after SGS preprocessing, the data processing results of other preprocessing methods are worse than the results of direct modeling of the original spectrum. In terms of the number of factors involved in modeling, the LVS of SGS in both modes increases by one, indicating that the modeling complexity has increased. However, the modeling accuracy of SGS is much higher than that of the original spectrum, so this effect is ignored. Compared with the original spectra, the *R*_*CV*_ of the model is increased by 0.160 and RMSECV is reduced by 0.413° Brix after the SGS pretreatment method is used for the static detection data. The *R*_*CV*_ is increased by 0.171 and RMSECV is reduced by 0.418° Brix after the SGS pretreatment method is used for online detection data.

#### Robust variable selection based on MCUVE method

**Figure 3:**
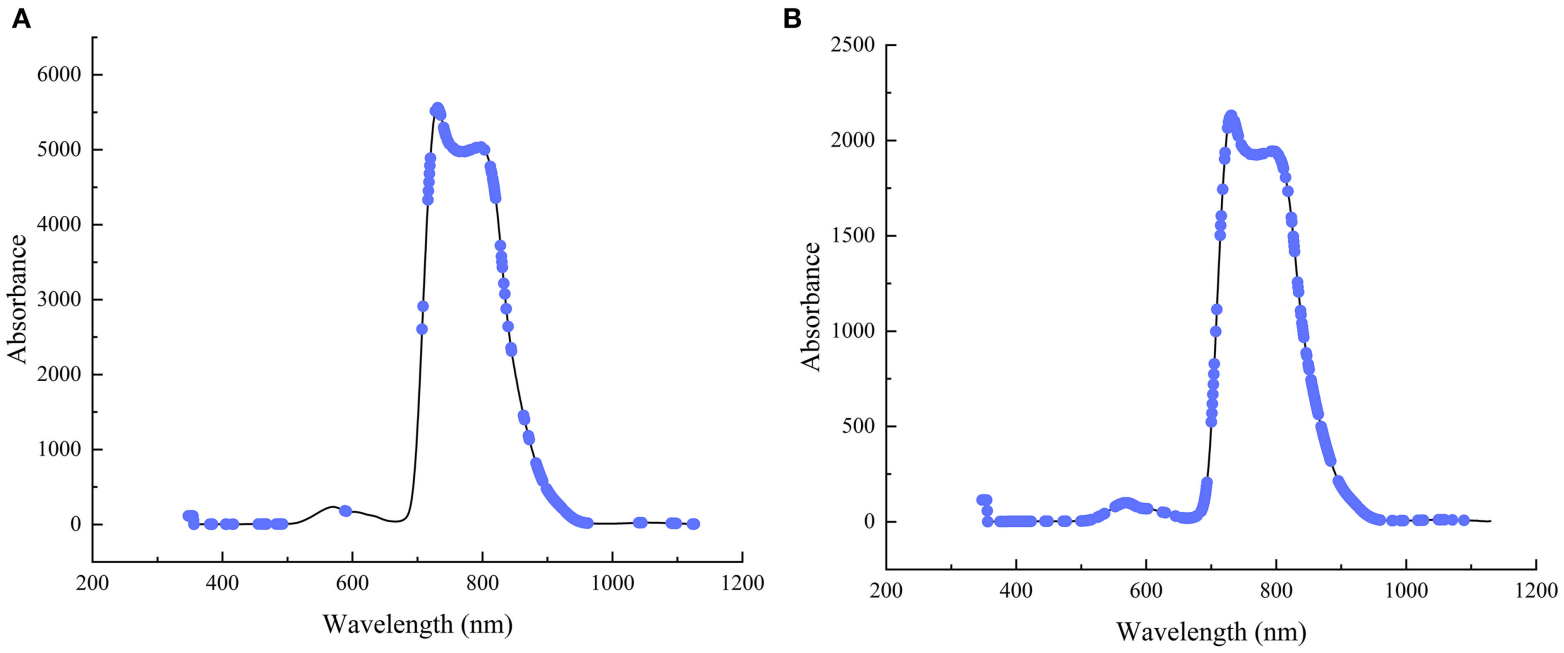
**(A)** Variable optimization results with MCUVE for static detection; **(B)** Variable optimization results with MCUVE for online detection.

In order to further improve the model accuracy, simplify the model and improve its interpretability, MCUVE method is used for wavelength selection based on SGS preprocessing. After the optimization, the wavelengths of static and online detection are reduced from 1,044 in a full spectrum to 250 and 450 respectively. The variable distribution optimized by MCUVE variable selection method for the two detection methods is shown in Figure 3. It can be seen from the figure that the selected modeling variables for static and online detection are mainly concentrated in three regions of 347–694, 716–820 and 827–1,129 nm. The selected variables in the spectral range of 347–694 nm are related to the absorption of carotenoids in snow peach (26); the selected variables in the spectral range of 716–820 nm are mainly related to the absorption of chlorophyll in snow peach (27); the selected variables in the spectral range of 827–1,129 nm are mainly related to the absorption of water in snow peach (28). It can be seen from Figure 3 that the regions of the variables selected by the MCUVE method for static and online detection are basically the same, indicating that the variable selection is reasonable.

### Construction of SSC robust model for snow peach

### Robust model construction and analysis based on SPLSR and SPRMR methods

Combined with SGS preprocessing algorithm and MCUVE variable selection algorithm, the robust models of SPLSR and SPRMR for the SSC of snow peach are established respectively. Table 3 shows the modeling results of snow peach SSC calibration set and prediction set based on different modeling methods. It can be seen from the table that the difference between *R*_*CV*_ and *R*_*P*_, RMSECV and RMSEP of the SPRMR model is smaller than that of the SPLSR model, indicating that the SPRMR model is more stable than the SPLSR model and its prediction results are more reliable. The RPD values of SPRMR and SPLSR in static detection are 5.926 and 5.498 respectively, which are >3, indicating that both models have excellent prediction performance in static detection mode. The RPD values of SPRMR and SPLSR in online detection are 3.848 and 3.744 respectively, indicating that both the SPRMR model and the SPLSR model have excellent prediction performance in online detection mode. In addition, the RER values of two robust regression models under different detection modes are all >10, and the RER value of static detection is higher than that of online detection.

**Table 3:** Quantitative analysis results of snow peach SSC with PLSR, SPLSR and SPRMR modeling methods.

**Models**	**Static**							**Online**						
	**Calibration set**			**Prediction set**				**Calibration set**			**Prediction set**			
	**LVs**	***R_*CV*_***	**RMSECV**	***R_*P*_***	**RMSEP**	**RPD**	**RER**	**LVs**	***R_*CV*_***	**RMSECV**	***R_*P*_***	**RMSEP**	**RPD**	**RER**
PLSR	18	0.992	0.124	0.983	0.182	5.263	26.981	19	0.991	0.128	0.966	0.247	3.800	20.245
SPLSR	18	0.991	0.131	0.984	0.174	5.498	28.191	19	0.993	0.109	0.965	0.251	3.744	19.944
SPRMR	19	0.994	0.106	0.987	0.161	5.926	30.388	19	0.993	0.111	0.967	0.244	3.848	20.500

### Reliability analysis of robust regression method with different background noise interference

**Figure 4:**
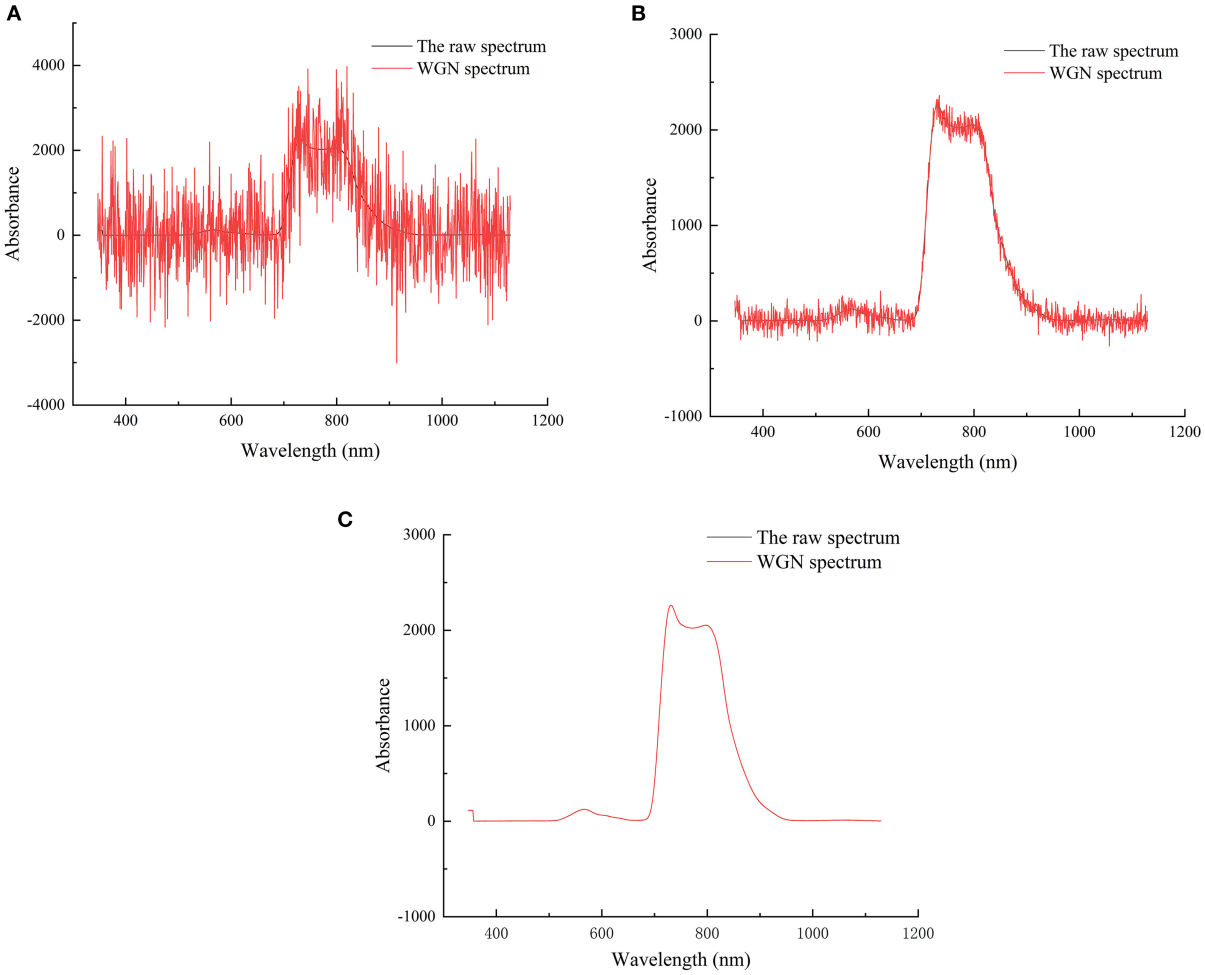
**(A)** Spectra with −10 SNR; **(B)** Spectra with 10 SNR; **(C)** Spectra with 50 SNR.

In order to test the robustness of the model, N random data in the calibration set samples and prediction set samples were selected from this experiment, and white gaussian noise (WGN) was added to observe the recognition accuracy of the PLSR, SPLSR and SPRMR models on noise and abnormal samples. In the experiment, the intensity of the added WGN is adjusted by changing the signal-to-noise ratio (SNR). N is set as 10, i.e., 10 samples are randomly drawn from both datasets and added with noise. Figure 4 shows three spectra with SNR of −10, 10, 50 in static detection mode.

**Figure 5:**
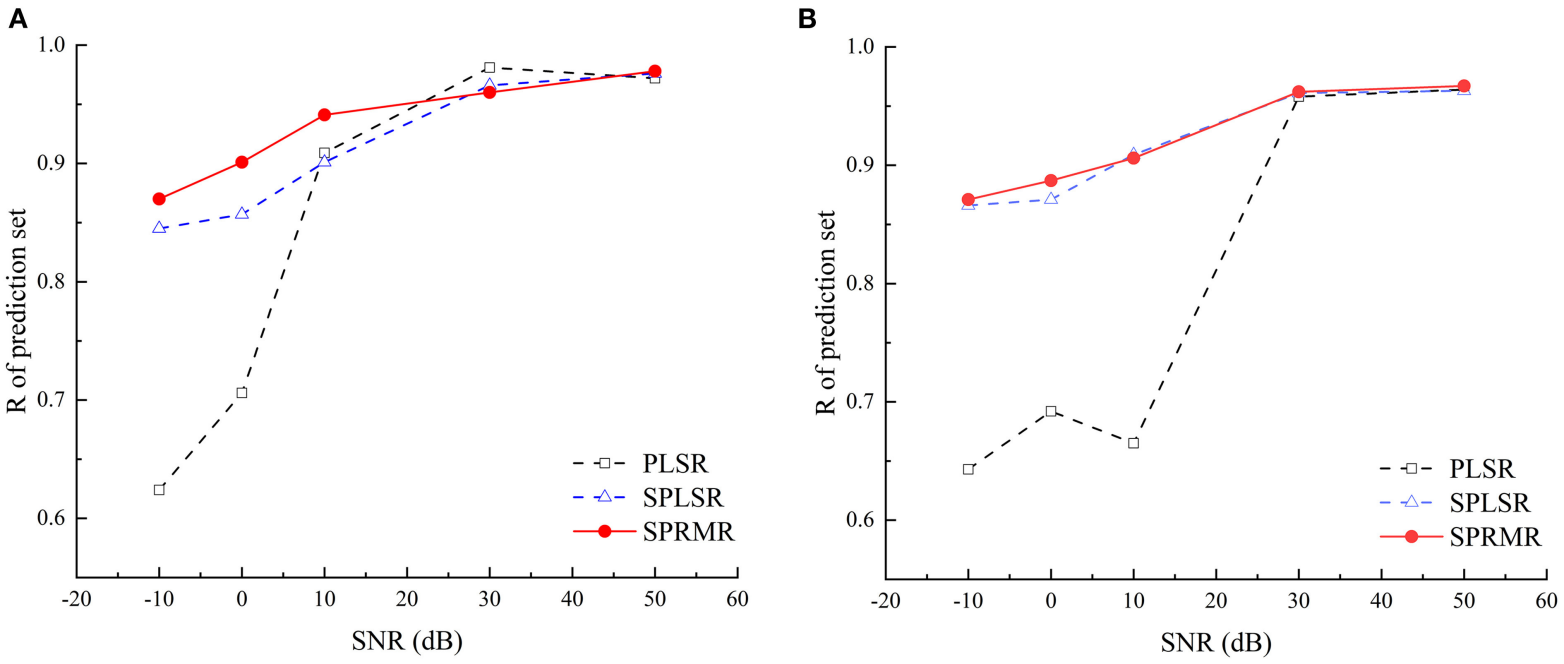
The influence trends of WGN with different SNR on calibration correlation coefficient of three models. **(A)** The static detection; **(B)** The online detection.

Generally, the larger the SNR is, the smaller the mixed noise is, so the model accuracy should gradually increase with the increase of SNR. The variation trend of *R*_*P*_ for different models with noise intensity is shown in Figure 5. It can be seen from Figure 5 that with the addition of different noises, the accuracy of SPLSR and SPRMR models is less affected; while the accuracy of PLSR model decreases greatly and shows instability. By observing the trend of the curve, it can be seen that the predictive ability of the SPRMR model is slightly higher than that of the SPLSR model. Even if the noise level reaches −10, the SPRMR model still has a good prediction performance and outperforms other models.

In order to verify the ability of the SPRMR model to automatically identify abnormal samples, the RMSEP of the data with noise, the data with non-noise and the whole data were calculated in the case of adding noise, respectively. The anti-interference ability of the SPRMR model is illustrated by observing the errors of the prediction set composed of two parts of data. Table 4 shows the RMSEP of the prediction set including 10 noise samples with SNR of 10. It can be seen from Table 4 that the prediction error of the SPRMR model for normal samples with non-noise is small, and the SSC of the samples can be more accurately predicted. However, the RMSEP of the data with noise exceeds 2° Brix, the deviation is larger. It shows that the noise data added in the training set can be accurately identified during the modeling of SPRMR. The model regards the samples with noise as abnormal samples, and assigns a smaller weight during modeling. So, for the 10 data with noise added in the prediction set, there is a larger prediction bias.

**Table 4:** The RMSEP of the prediction set including 10 noise samples with SNR of 10.

**Models**	**RMSEP of static detection**			**RMSEP of online detection**		
	**Noise**	**Non-noise**	**Whole**	**Noise**	**Non-noise**	**Whole**
PLSR	1.148	0.738	0.776	0.806	0.722	0.729
SPLSR	0.551	0.556	0.556	0.659	0.452	0.470
SPRMR	2.241	0.271	0.667	2.065	0.366	0.666

For another robust regression model, SPLSR, it can be observed from Table 4 that there is little difference between the RMSEP results of the data with noise and the data with non-noise. This is because the SPLSR model does not have the ability to identify outliers, and the abnormal samples are regarded as normal samples during the modeling process and participate in the modeling. In addition, the model has inherent variable selection property, which can automatically discard the variables that have a greater impact on the modeling results during modeling, and optimize the model to a certain extent. Therefore, the RMSEP of the data with noise and the data with non-noise are basically similar.

In conclusion, robust regression models are less affected by noise, and have the advantages of preventing over-fitting and reducing the influence of outliers or strong influence values on the stability of the model. In addition, the SPRMR model can identify outliers and has strong anti-interference ability.

### Analysis of SSC optimal robust model for snow peach

A suitable robust regression model is established to achieve a better prediction of snow peach SSC in static and online detection. Through values of *R*_*P*_, RMSEP, RPD and RER, the PLSR, SPLSR and SPRMR models are comprehensively compared. It can be seen from Table 3 that the analytical model with the best prediction of snow peach SSC content is SPRMR for both static and online detection. Since the experimental samples are collected from the same orchard and the interference and noise contained in the sample information are small, the superior performance of the SPRMR model has not been fully highlighted. However, it can be seen from the noise experiment in the previous section that the SPRMR model is still superior to the PLSR and SPLSR models in the presence of strong noises, showing strong anti-interference ability. Therefore, the optimal model for snow peach SSC prediction is the SPRMR model combined with SGS and MCUVE.

**Figure 6:**
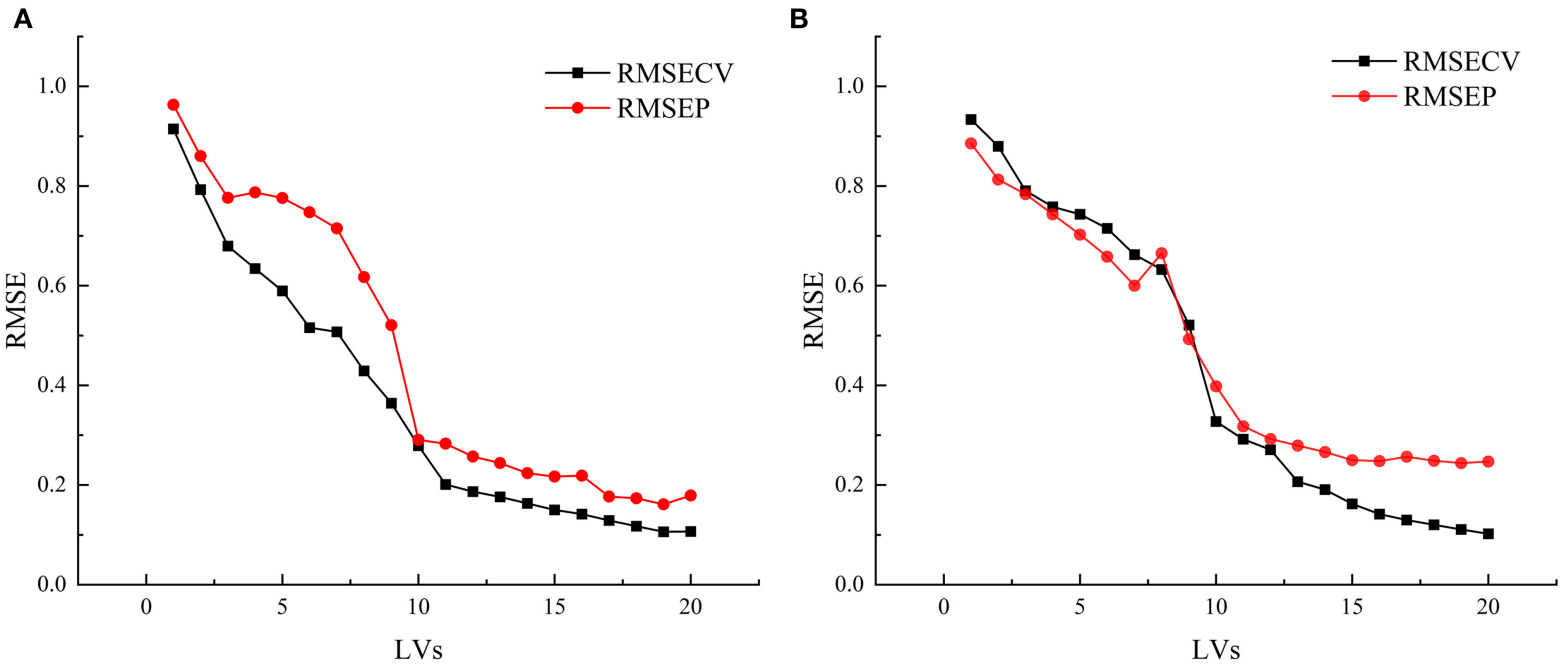
**(A)** Factor graph of SPRMR model for static detection; **(B)** Factor graph of SPRMR model for online detection.

**Figure 7:**
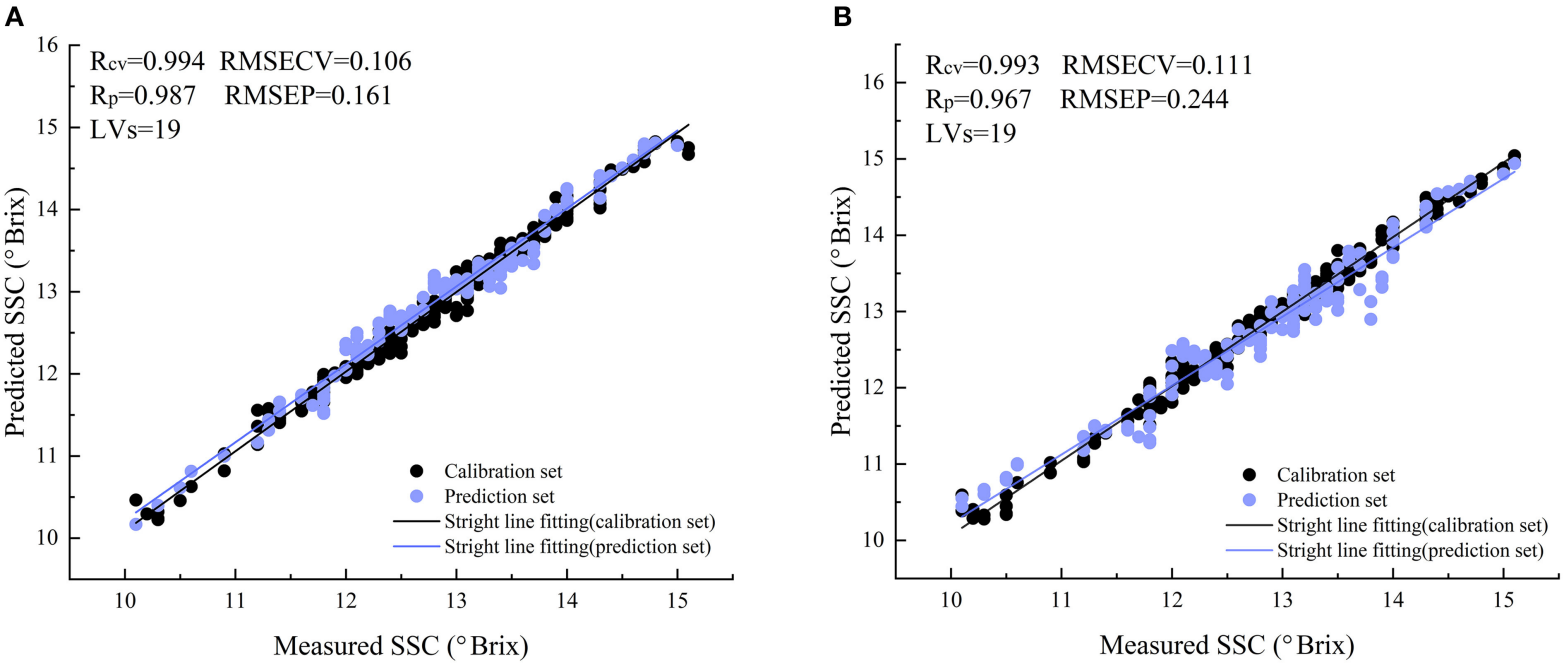
The correlation diagrams between the measured values of SSC and the predicted values of the models. **(A)** The static detection of snow peach; **(B)** The online detection of snow peach.

Figure 6 shows the factor diagram of the variation of RMSECV and RMSEP with the principal component fraction of the SPRMR model. The number of factors in both static and online models is 19. It can be seen from the figure that the variation trends of RMSECV and RMSEP are basically similar, indicating that the model fitting is reasonable. Figure 7 shows the correlation between the measured values and predicted values of snow peach SSC. It can be seen from the figure that for the static detection model, when the factor number is 19, the *R*_*CV*_ and *R*_*P*_ of the model are 0.994 and 0.987 respectively, and RMSECV and RMSEP are 0.106 and 0.161 respectively. For the online model, when the factor number is 19, the *R*_*CV*_ and *R*_*P*_ are 0.993 and 0.967 respectively, and RMSECV and RMSEP are 0.111 and 0.244 respectively. The analysis shows that the correlation coefficient (R) and Root Mean Square Error (RMSE) under two detection modes are similar, indicating that the over-fitting risk of the model is small. In general, the SPRMR model has strong anti-noise ability, which can realize the reliable analysis of snow peach SSC.

## Conclusion

The Vis-NIR spectroscopy combined with PLSR, SPLSR and SPRMR methods is used for rapid analysis of SSC in snow peach. NWS, SGS, CWD, MSC and VSN preprocessing methods and MCUVE variable selection method are used to enhance and optimize the information of snow peach characteristic variables. The results show that the SGS preprocessing method can effectively improve the analysis accuracy of static and online models. The MCUVE method realizes the extraction of stable characteristic variables; the accuracy of static detection is slightly better than that of online detection. The SPRMR method has strong robustness and anti-interference ability. Therefore, the SPRMR model constructed by combining Vis-NIR spectroscopy combined with SGS preprocessing method and MCUVE variable selection method can realize the prediction of SSC in snow peach.

In view of the current research on single system of static detection or online detection, this experiment makes a comparative analysis of the two detection methods. The application of a robust model in fruit SSC has been discussed, which provides ideas for the future application of robust models combined with Vis-NIR spectroscopy in fruit quality detection. The shortcomings of this experiment are as follows: (1) Although 400 experimental samples are collected in batches from different planting areas of the same orchard, the time interval is so short that sample spectra do not contain information about different planting environments; (2) In the Vis-NIR spectrum acquisition, the peaches are not fully mature and their skin is hard (if analyzed after maturity, it is easy to bump in the grading process). In this case, the SSC detection value is small, and the distribution of SSC value affects the subsequent modeling accuracy.

## Data availability statement

The raw data supporting the conclusions of this article will be made available by the authors, without undue reservation.

## Author contributions

YH: conceptualization, investigation, supervision, and writing—original draft and review and editing. XL: methodology, formal analysis, software, and writing—original draft and editing. CZ: formal analysis and writing—original draft and review. ZL: supervision, conceptualization, and writing—review and editing. All authors contributed to the article and approved the submitted version.

## Funding

This study was funded by the National Natural Science Foundation of China (grant number 31960497), Jiangxi Provincial Natural Science Foundation of China (grant number 20212BAB204009), and Jiangxi Provincial Natural Science Foundation of China (grant number 20202ACB211002).

## Conflict of interest

The authors declare that the research was conducted in the absence of any commercial or financial relationships that could be construed as a potential conflict of interest.

## Publisher's note

All claims expressed in this article are solely those of the authors and do not necessarily represent those of their affiliated organizations, or those of the publisher, the editors and the reviewers. Any product that may be evaluated in this article, or claim that may be made by its manufacturer, is not guaranteed or endorsed by the publisher.
